# The role of microbiota in immunotherapy outcomes in colorectal cancer patients: A protocol for a systematic review

**DOI:** 10.1371/journal.pone.0273314

**Published:** 2022-08-19

**Authors:** Suad Ajab, Sumaya Zoughbor, Lena Labania, Marie Olanda, Linda Östlundh, Zakeya Al Rasbi

**Affiliations:** 1 Institute of Public Health, College of Medicine and Health Sciences, United Arab Emirates University, Al Ain, Abu Dhabi, United Arab Emirates; 2 Microbiology and Immunology, College of Medicine and Health Sciences, United Arab Emirates University, Al Ain, Abu Dhabi, United Arab Emirates; 3 National Medical Library, United Arab Emirates University, Al Ain, Abu Dhabi, United Arab Emirates; Hamad Medical Corporation, QATAR

## Abstract

In the human gut, there are many microbes, including bacteria, viruses and parasites. The imbalance in the numbers of each type of these microbes can translate into gastrointestinal disorders. Lately, different microbiota patterns have been associated with the levels of efficacy of immunotherapy in multiple cancer conditions. Studies have shown that patients with a more diverse gut microbiome respond better to immunotherapy than those with a homogeneous microbiome. This systematic review aims to identify and assess the available evidence on the efficacy of immunotherapy in treating colorectal cancer (CRC) patients and the effect of their microbiota on their treatment outcomes. The researchers will study the literature regarding CRC and immunotherapy outcomes to survey the different approaches employed to assess the treatment outcomes. A systematic search will be performed in five biomedical databases (PubMed, Scopus, Web of Science, Embase, and the Cochrane Library) in June-July, 2022. Also, open-access registers of clinical trials will be trawled. The search will be conducted without geographical or publication date restrictions; however, only papers published in the English language will be sought. Details regarding patients’ diets, lifestyles, and characteristics will be assessed. We will define the primary outcome to compare CRC patients’ immunotherapy responses with their gut microbiota composition. The systematic review methodology does not require ethics approval due to the nature of the study design. The systematic review results will be published in an open-access peer-reviewed journal.

**PROSPERO ID**: CRD42021277691.

## Introduction

### Background

Chronic diseases, such as cancer, are among the leading causes of worldwide death [1]. Globally, colorectal cancer (CRC) is the third most prevalent cancer and caused the second-highest number of cancer deaths in 2018 [2]. Gut microbiota play a vital role in CRC prognosis [3]. Many studies have reported an association between gut microbiota including bacteria, archaea, viruses, fungi and protozoa, and cancer treatment outcomes [1]. Immunotherapy is used against cancer by boosting the immune system of individuals [4]. It has been reported that gut microbiota may influence the response of individuals to cancer immunotherapy [5]. In this paper, we present the protocol for a systematic review of human observational studies that have considered the role of microbiota in CRC immunotherapy outcomes.

### Description of the exposure: The gut microbiota

There is increasing evidence that our gut microbiota can profoundly impact our overall health and response to disease [6, 7]. Normal human gut microbiota comprise two significant phyla: *Bacteroidetes* and *Firmicutes* [6]. The contents of gut microbiota are influenced by many factors that include the location where the individual resides, whether or not they smoke, their diet and medications [7, 8]. Development of some gastrointestinal malignancies, such as CRC, has been associated with dysbiosis, an imbalance in the microflora that make up the gut microbiota [6–8]. Nevertheless, a favorable response has been observed across cohort studies when specific bacterial taxa were in high load in therapy "responders" compared with "non-responders" [9, 10].

### Description of the outcome: Cancer immunotherapy

Immune checkpoint inhibitors are a type of cancer immunotherapy that restores and activates the immune system. Examples include antibodies that block the cytotoxic T-lymphocyte-associated protein 4 (CTLA-4) (ipilimumab); antagonists to programmed cell death protein 1 (PD-1) (pembrolizumab and nivolumab); and inhibitors of programmed death ligand 1 (PD-L1) (atezolizumab, avelumab, and durvalumab) [11]. Nivolumab and pembrolizumab act as checkpoint inhibitors that target the PD-1/PD-L1 pathway in CRC [12]. Many studies have demonstrated that gut microbiota modulates the responses to immunotherapy across several cancer types [8].

### The rationale for a systematic review

There is compelling evidence that the contents of gut microbiota affect cancer immunotherapy outcomes [9, 10, 13, 14]. The effect has been assessed in several experimental studies [8–10]. Independently of pathogenesis, enhancing the gut microbiota through the introduction of certain probiotics may increase the overall survival rates of CRC patients who receive immunotherapy. If this can be confirmed, this would lead to new recommendations in cancer clinical practice.

This systematic review will influence future cancer research regarding treatment strategies and precision medicine.

### Objectives

This systematic review aims to study the role of microbiota in immunotherapy outcomes in CRC patients. The findings of this systematic review may guide clinical practice to improve cancer patient care and stimulate the performance of additional studies regarding the influence of microbiota on cancer treatment options.

## Methods and analysis

This systematic review protocol will follow the preferred reporting items for systematic reviews and meta-analysis protocols (PRISMA-P) in S1 Checklist, defined in 2015 [15]. The protocol has been registered to the international prospective register of systematic reviews platform (PROSPERO) with registration ID: CRD42021277691.

### Eligibility criteria

The studies to be included will conform to the population, intervention, comparison, and outcome (PICO) model, as described by the PRISMA statement.

### Type of population

We will include studies of patients of any age who have CRC, are receiving immunotherapy, and whose gut microbiota composition is reported using molecular techniques.

### Type of intervention

Primary studies that report immunotherapy results on CRC patients will be included. We will evaluate details regarding patients’ characteristics, diets, lifestyles, and the duration of immunotherapy and stage of CRC (stable, unstable, deteriorating, terminal).

### Type of comparator

The comparator item will be the gut microbiota composition in CRC patients receiving immunotherapy.

### Type of outcomes

We have defined the primary outcome as the immunotherapy response, reported as progression-free or overall survival.

### Type of studies

We will consider published, peer-reviewed epidemiological studies, and clinical trials registry electronic databases on CRC patients who have been exposed to immunotherapy and whose gut microbiota composition is reported according to molecular analysis.

### Information sources

Electronic biomedical databases: PubMed, Scopus, Web of Science, Embase, and the Cochrane Library will be searched, as well as open access registers of clinical trials.

### Search strategy

We performed systematic pre-searches in PubMed in March-April 2022 to identify a relevant search strategy. The search terms were determined based on the population, intervention, comparator, and outcomes (PICO) model and systematically developed with the help of PubMed’s medical subject headings (MeSH). In the final search, all search terms will be used in a combination of the search “Text word”, “Title/Abstract”, and MeSH (if applicable) without any publication year or geographical restrictions during June and July 2022. A filter for English language will be applied.

The search strategy defined in PubMed will be adapted for use in and systematically repeated in all selected information sources. Reference lists of previously published reviews identified in the search and in the selected studies will be hand-screened for potential additional, eligible studies. Reproducible and transparent search documentation of all information sources, including the technical details of the search, the results, and notes, will be appended to the final review.

### Data statement

The pre-search, which was performed in PubMed on 28 June 2022, is shown in S1 Appendix.

### Data management

Records identified in the database search will be uploaded to Covidence, which is a systematic review software that will assess in the automatic removal of duplicates and blinded screening and extraction [16].

### Selection process

Two independent reviewers will use Covidence to screen the titles and abstracts of all unique records that are identified in the literature search against the pre-set inclusion and exclusion criteria. Disagreements will be resolved by a third reviewer not involved in screening. Via Covidence, the full texts of the studies that are identified as potentially eligible through the title and abstract screening will be reviewed by two independent reviewers. A third reviewer will resolve any disagreements.

### Data collection process

Upon the authors’ agreement, a pre-defined data extraction sheet will be tailored to capture all variables relevant to the primary immunotherapy outcome and the main comparator, the gut microbiota.

Using the conflict resolution function in Covidence, two or more researchers will extract the data. A third researcher will review the extracted information and resolve any differences presented automatically by the software.

In addition, eligible studies’ authors will be contacted via e-mail if any additional or missing data are needed.

### Data items

The extracted data will comprise of firstly essential publication characteristics such as title, name of the first author, date of publication, and digital object identifier (DOI); secondly, study design including study type, study population, period, country, measurement of exposure, prognostic factors and outcome, and adjustment for confounders; and lastly, results such as gut microbiota composition and therapy outcome.

### Outcomes and prioritization

We will extract data reported on microbiota composition upon treatment with immunotherapy. We will not apply prioritization schemes as no meta-analysis will be performed. All the relevant data will be extracted as string variables.

### Risk of bias in individual studies

Two independent reviewers will assess the quality of the evidence, any conflicts of interest in terms of the declaration or funding, and the risk of bias in the eligible studies. They will use the study quality assessment tools of the National Institutes of Health. A third reviewer will resolve any disagreement. Any further discrepancies will be checked with the study investigator, and the quality of the included studies will be detailed. We will not exclude studies based on their quality report due to the limited number of published, peer-reviewed studies investigating the impact of microbiota on cancer treatment outcomes.

### Data synthesis

The results regarding the primary outcome will be summarised in tables. These tables will contain the year of publication, the country in which the study was performed, the sample population, and the type of immunotherapy. A descriptive synthesis of the findings will be transcribed from the eligible studies, structured from the patients’ characteristics and the type of CRC immunotherapy management. We will summarise the role of microbiota in immunotherapy outcomes in CRC patients that was found in each study.

### Analysis of subgroups or subsets

Subgroup analysis will be performed by age groups, gender and study design. Sensitivity analysis will be conducted, including studies judged to be of "low" or "probably low" risk of general bias or conflict-of-interest bias.

### Patient and public involvement

Neither patients nor the public will be involved in this study.

### Ethics and dissemination

Ethics approval for the current systematic review methodology is not required; due to the nature of the study design. The systematic review results will be published in an open-access peer-reviewed journal, and the review will be disseminated electronically and in printed versions.

## Supporting information

S1 Checklist PRISMA-P 2015 checklist: Recommended items to address in a systematic review protocol. (DOCX)

S1 Appendix Pre search for: The role of microbiota in immunotherapy outcomes in colorectal cancer patients. (DOCX)
