# Supplementary material for: The role of microbiota in immunotherapy outcomes in colorectal cancer patients: A protocol for a systematic review
Source: PLoS One. 2022 Aug 19;17(8):e0273314. doi: 10.1371/journal.pone.0273314 (PMC9390889; doi:10.1371/journal.pone.0273314)
Supplement: S1 Appendix — (DOCX) [file pone.0273314.s002.docx]

**Pre search for: The role of microbiota in immunotherapy outcomes in colorectal cancer patients**

**Source:** PubMed

**Search date:** 2022-06-28

**Basic search string:** “colon neoplasm” AND Microbiome AND “immune therapy”

| **Search no. 1** | **Search String (Search terms)** | **Results** | **Notes** |
| --- | --- | --- | --- |
|  | "colonic neoplasm*"[Title/Abstract] OR "colon neoplasm*"[Title/Abstract] OR "Cancer of Colon*"[Title/Abstract] OR "Colon Cancer*"[Title/Abstract] OR "Colonic Cancer*"[Title/Abstract] OR "aberrant crypt foc*" [Title/Abstract] OR "colorectal neoplasm*"[Title/Abstract] OR "Colorectal Tumor*"[Title/Abstract] OR "Colorectal Cancer*"[Title/Abstract] OR "Colorectal Carcinoma*"[Title/Abstract] OR "colonic polyp*"[Title/Abstract] OR "colitis associated neoplasm*"[Title/Abstract] OR "digestive system neoplasm*"[Title/Abstract] OR "Cancer of Digestive System*"[Title/Abstract] OR "Digestive System Cancer*"[Title/Abstract] OR "colonic neoplasm*"[Text Word] OR "colon neoplasm*"[Text Word] OR "Cancer of Colon*"[Text Word] OR "Colon Cancer*"[Text Word] OR "Colonic Cancer*"[Text Word] OR "aberrant crypt foc*" [Text Word] OR "colorectal neoplasm*"[Text Word] OR "Colorectal Tumor*"[Text Word] OR "Colorectal Cancer*"[Text Word] OR "Colorectal Carcinoma*"[Text Word] OR "colonic polyp*"[Text Word] OR "colitis associated neoplasm*"[Text Word] OR "digestive system neoplasm*"[Text Word] OR "Cancer of Digestive System*"[Text Word] OR "Digestive System Cancer*"[Text Word] OR "Colonic Neoplasms"[Mesh] OR "Aberrant Crypt Foci"[Mesh] OR "Colorectal Neoplasms"[Mesh] OR "Colonic Polyps"[Mesh] OR "Digestive System Neoplasms"[Mesh] OR "sigmoid neoplasm*"[Title/Abstract] OR "Intestinal Neoplasm*"[Title/Abstract] OR "Intestines Neoplasm*"[Title/Abstract] OR "Intestine Neoplasm*"[Title/Abstract] OR "Intestine cancer**"[Title/Abstract] OR "Cancer of Intestine*"[Title/Abstract] OR "Intestines Cancer*"[Title/Abstract] OR "Intestine Cancer*"OR "Cancer of the Intestine*"[Title/Abstract] OR "Intestinal Cancer*"[Title/Abstract] OR "sigmoid neoplasm*"[Text Word] OR "Intestinal Neoplasm*"[Text Word] OR "Intestines Neoplasm*"[Text Word] OR "Intestine Neoplasm*"[Text Word] OR "Intestine cancer**"[Text Word] OR "Cancer of Intestine*"[Text Word] OR "Intestines Cancer*"[Text Word] OR "Intestine Cancer*"OR "Cancer of the Intestine*"[Text Word] OR "Intestinal Cancer*"[Text Word] OR "Intestinal Neoplasms"[Mesh] OR "Gastrointestinal Neoplasms"[Mesh] OR "gastrointestinal neoplasm*"[Title/Abstract] OR "Cancer of Gastrointestinal Tract*"[Title/Abstract] OR "Gastrointestinal Tract Cancer*"[Title/Abstract] OR "Cancer of the Gastrointestinal Tract"[Title/Abstract] OR "Gastrointestinal Cancer*"[Title/Abstract] OR "gastrointestinal neoplasm*"[Text Word] OR "Cancer of Gastrointestinal Tract*"[Text Word] OR "Gastrointestinal Tract Cancer*"[Text Word] OR "Cancer of the Gastrointestinal Tract"[Text Word] OR "Gastrointestinal Cancer*"[Text Word] | **625,994** | Search on: “Colonic Neoplasm*” and synonyms  Notes:  All keywords are searched in the fields: “Text word”, “title” and “abstract”, and in MeSH when available.  The search is limited to English studies only. |
| **Search no. 2** | "Gastrointestinal Microbiome*"[MeSH] OR "Gastrointestinal Microbiome*"[Text Word] OR "Gut Microbiome*"[Text Word] OR "Gut Microflora"[Text Word] OR "Gut Microbiota*"[Text Word] OR "Gastrointestinal Flora"[Text Word] OR "Gut Flora"[Text Word] OR "Gastrointestinal Microb*"[Text Word] OR "Gastrointestinal Microbial Communit*"[Text Word] OR "Gastrointestinal Microflora"[Text Word] OR "Gastric Microbiome*"[Text Word] OR "Intestinal Microbiome*"[Text Word] OR "Intestinal Microbiota*"[Text Word] OR "Intestinal Microflora"[Text Word] OR "Intestinal Flora"[Text Word] OR "Enteric Bacteria*"[Text Word] OR "Gastrointestinal Microbiome*"[Title/Abstract] OR "Gut Microbiome*"[Title/Abstract] OR "Gut Microflora"[Title/Abstract] OR "Gut Microbiota*"[Title/Abstract] OR "Gastrointestinal Flora"[Title/Abstract] OR "Gut Flora"[Title/Abstract] OR "Gastrointestinal Microb*"[Title/Abstract] OR "Gastrointestinal Microbial Communit*"[Title/Abstract] OR "Gastrointestinal Microflora"[Title/Abstract] OR "Gastric Microbiome*"[Title/Abstract] OR "Intestinal Microbiome*"[Title/Abstract] OR "Intestinal Microbiota*"[Title/Abstract] OR "Intestinal Microflora"[Title/Abstract] OR "Intestinal Flora"[Title/Abstract] OR "Enteric Bacteria*"[Title/Abstract] | **67,744** | Search on:  Gut  Microbiome and synonyms  Notes:  All keywords are searched in the fields: “Text word”, “title” and “abstract”, and in MeSH when available.  The search is limited to English studies only. |
| **Search no. 3** | "Immunotherapy"[Mesh] OR "Biological Therapy"[Mesh] OR "Immune Checkpoint Inhibitors"[Mesh] OR "Immunotherp*"[Text Word] OR "Biologic* Therap*"[Text Word] OR "Biotherap*"[Text Word] OR "Adoptive Immunotherap*"[Text Word] OR "Adoptive Cellular Immunotherap*"[Text Word] OR "Chimeric Antigen Receptor Therap*"[Text Word] OR "CAR T-Cell Therap*"[Text Word] OR "CAR T Cell Therap*"[Text Word] OR "Immune Checkpoint Inhibit*"[Text Word] OR "Immune Checkpoint Block*"[Text Word] OR "PD-L1 Inhibit*"[Text Word] OR "PD L1 Inhibit*"[Text Word] OR "Programmed Death-Ligand 1 Inhibit*"[Text Word] OR "Programmed Death Ligand 1 Inhibit*"[Text Word] OR "CTLA-4 Inhibit*"[Text Word] OR "CTLA 4 Inhibit*"[Text Word] OR "Cytotoxic T-Lymphocyte-Associated Protein 4 Inhibit*"[Text Word] OR "Cytotoxic T Lymphocyte Associated Protein 4 Inhibit*"[Text Word] OR "PD-1 Inhibit*"[Text Word] OR "PD 1 Inhibit*"[Text Word] OR "Programmed Cell Death Protein 1 Inhibit*"[Text Word] OR "PD-1-PD-L1 Block*"[Text Word] OR "PD 1 PD L1 Block*"[Text Word] OR "Anti-PD-1"[Text Word] OR "Anti PD 1"[Text Word] OR "Anti-PD-L1"[Text Word] OR "Anti PD L1"[Text Word] OR "Anti-CTLA-4"[Text Word] OR "Anti CTLA 4"[Text Word] OR "Anti‐cytotoxic T‐lymphocyte‐associated protein 4"[Text Word] OR "Pembrolizumab"[Text Word] OR "Keytruda" [Text Word] OR "Opdivo"[Text Word] OR "Nivolumab"[Text Word] OR "Ipilimumab"[Text Word] OR "Immunotherp*"[Title/Abstract] OR "Biologic* Therap*"[Title/Abstract] OR "Biotherap*"[Title/Abstract] OR "Adoptive Immunotherap*"[Title/Abstract] OR "Adoptive Cellular Immunotherap*"[Title/Abstract] OR "Chimeric Antigen Receptor Therap*"[Title/Abstract] OR "CAR T-Cell Therap*"[Title/Abstract] OR "CAR T Cell Therap*"[Title/Abstract] OR "Immune Checkpoint Inhibit*"[Title/Abstract] OR "Immune Checkpoint Block*"[Title/Abstract] OR "PD-L1 Inhibit*"[Title/Abstract] OR "PD L1 Inhibit*"[Title/Abstract] OR "Programmed Death-Ligand 1 Inhibit*"[Title/Abstract] OR "Programmed Death Ligand 1 Inhibit*"[Title/Abstract] OR "CTLA-4 Inhibit*"[Title/Abstract] OR "CTLA 4 Inhibit*"[Title/Abstract] OR "Cytotoxic T-Lymphocyte-Associated Protein 4 Inhibit*"[Title/Abstract] OR "Cytotoxic T Lymphocyte Associated Protein 4 Inhibit*"[Title/Abstract] OR "PD-1 Inhibit*"[Title/Abstract] OR "PD 1 Inhibit*"[Title/Abstract] OR "Programmed Cell Death Protein 1 Inhibit*"[Title/Abstract] OR "PD-1-PD-L1 Block*"[Title/Abstract] OR "PD 1 PD L1 Block*"[Title/Abstract] OR "Anti-PD-1"[Title/Abstract] OR "Anti PD 1"[Title/Abstract] OR "Anti-PD-L1"[Title/Abstract] OR "Anti PD L1"[Title/Abstract] OR "Anti-CTLA-4"[Title/Abstract] OR "Anti CTLA 4"[Title/Abstract] OR "Anti‐cytotoxic T‐lymphocyte‐associated protein 4"[Title/Abstract] OR "Pembrolizumab"[Title/Abstract] OR "Keytruda" [Title/Abstract] OR "Opdivo"[Title/Abstract] OR "Nivolumab"[Title/Abstract] OR "Ipilimumab"[Title/Abstract] | **683,283** | Search on: Immune therapy and synonyms  Notes:  All keywords are searched in the fields: “Text word”, “title” and “abstract”, and in MeSH when available.  The search is limited to English studies only. |
| **Search no. 4** | (("colonic neoplasm*"[Title/Abstract] OR "colon neoplasm*"[Title/Abstract] OR "Cancer of Colon*"[Title/Abstract] OR "Colon Cancer*"[Title/Abstract] OR "Colonic Cancer*"[Title/Abstract] OR "aberrant crypt foc*" [Title/Abstract] OR "colorectal neoplasm*"[Title/Abstract] OR "Colorectal Tumor*"[Title/Abstract] OR "Colorectal Cancer*"[Title/Abstract] OR "Colorectal Carcinoma*"[Title/Abstract] OR "colonic polyp*"[Title/Abstract] OR "colitis associated neoplasm*"[Title/Abstract] OR "digestive system neoplasm*"[Title/Abstract] OR "Cancer of Digestive System*"[Title/Abstract] OR "Digestive System Cancer*"[Title/Abstract] OR "colonic neoplasm*"[Text Word] OR "colon neoplasm*"[Text Word] OR "Cancer of Colon*"[Text Word] OR "Colon Cancer*"[Text Word] OR "Colonic Cancer*"[Text Word] OR "aberrant crypt foc*" [Text Word] OR "colorectal neoplasm*"[Text Word] OR "Colorectal Tumor*"[Text Word] OR "Colorectal Cancer*"[Text Word] OR "Colorectal Carcinoma*"[Text Word] OR "colonic polyp*"[Text Word] OR "colitis associated neoplasm*"[Text Word] OR "digestive system neoplasm*"[Text Word] OR "Cancer of Digestive System*"[Text Word] OR "Digestive System Cancer*"[Text Word] OR "Colonic Neoplasms"[Mesh] OR "Aberrant Crypt Foci"[Mesh] OR "Colorectal Neoplasms"[Mesh] OR "Colonic Polyps"[Mesh] OR "Digestive System Neoplasms"[Mesh] OR "sigmoid neoplasm*"[Title/Abstract] OR "Intestinal Neoplasm*"[Title/Abstract] OR "Intestines Neoplasm*"[Title/Abstract] OR "Intestine Neoplasm*"[Title/Abstract] OR "Intestine cancer**"[Title/Abstract] OR "Cancer of Intestine*"[Title/Abstract] OR "Intestines Cancer*"[Title/Abstract] OR "Intestine Cancer*"OR "Cancer of the Intestine*"[Title/Abstract] OR "Intestinal Cancer*"[Title/Abstract] OR "sigmoid neoplasm*"[Text Word] OR "Intestinal Neoplasm*"[Text Word] OR "Intestines Neoplasm*"[Text Word] OR "Intestine Neoplasm*"[Text Word] OR "Intestine cancer**"[Text Word] OR "Cancer of Intestine*"[Text Word] OR "Intestines Cancer*"[Text Word] OR "Intestine Cancer*"OR "Cancer of the Intestine*"[Text Word] OR "Intestinal Cancer*"[Text Word] OR "Intestinal Neoplasms"[Mesh] OR "Gastrointestinal Neoplasms"[Mesh] OR "gastrointestinal neoplasm*"[Title/Abstract] OR "Cancer of Gastrointestinal Tract*"[Title/Abstract] OR "Gastrointestinal Tract Cancer*"[Title/Abstract] OR "Cancer of the Gastrointestinal Tract"[Title/Abstract] OR "Gastrointestinal Cancer*"[Title/Abstract] OR "gastrointestinal neoplasm*"[Text Word] OR "Cancer of Gastrointestinal Tract*"[Text Word] OR "Gastrointestinal Tract Cancer*"[Text Word] OR "Cancer of the Gastrointestinal Tract"[Text Word] OR "Gastrointestinal Cancer*"[Text Word] AND (english[Filter])) AND ("Immunotherapy"[Mesh] OR "Biological Therapy"[Mesh] OR "Immune Checkpoint Inhibitors"[Mesh] OR "Immunotherp*"[Text Word] OR "Biologic* Therap*"[Text Word] OR "Biotherap*"[Text Word] OR "Adoptive Immunotherap*"[Text Word] OR "Adoptive Cellular Immunotherap*"[Text Word] OR "Chimeric Antigen Receptor Therap*"[Text Word] OR "CAR T-Cell Therap*"[Text Word] OR "CAR T Cell Therap*"[Text Word] OR "Immune Checkpoint Inhibit*"[Text Word] OR "Immune Checkpoint Block*"[Text Word] OR "PD-L1 Inhibit*"[Text Word] OR "PD L1 Inhibit*"[Text Word] OR "Programmed Death-Ligand 1 Inhibit*"[Text Word] OR "Programmed Death Ligand 1 Inhibit*"[Text Word] OR "CTLA-4 Inhibit*"[Text Word] OR "CTLA 4 Inhibit*"[Text Word] OR "Cytotoxic T-Lymphocyte-Associated Protein 4 Inhibit*"[Text Word] OR "Cytotoxic T Lymphocyte Associated Protein 4 Inhibit*"[Text Word] OR "PD-1 Inhibit*"[Text Word] OR "PD 1 Inhibit*"[Text Word] OR "Programmed Cell Death Protein 1 Inhibit*"[Text Word] OR "PD-1-PD-L1 Block*"[Text Word] OR "PD 1 PD L1 Block*"[Text Word] OR "Anti-PD-1"[Text Word] OR "Anti PD 1"[Text Word] OR "Anti-PD-L1"[Text Word] OR "Anti PD L1"[Text Word] OR "Anti-CTLA-4"[Text Word] OR "Anti CTLA 4"[Text Word] OR "Anti‐cytotoxic T‐lymphocyte‐associated protein 4"[Text Word] OR "Pembrolizumab"[Text Word] OR "Keytruda" [Text Word] OR "Opdivo"[Text Word] OR "Nivolumab"[Text Word] OR "Ipilimumab"[Text Word] OR "Immunotherp*"[Title/Abstract] OR "Biologic* Therap*"[Title/Abstract] OR "Biotherap*"[Title/Abstract] OR "Adoptive Immunotherap*"[Title/Abstract] OR "Adoptive Cellular Immunotherap*"[Title/Abstract] OR "Chimeric Antigen Receptor Therap*"[Title/Abstract] OR "CAR T-Cell Therap*"[Title/Abstract] OR "CAR T Cell Therap*"[Title/Abstract] OR "Immune Checkpoint Inhibit*"[Title/Abstract] OR "Immune Checkpoint Block*"[Title/Abstract] OR "PD-L1 Inhibit*"[Title/Abstract] OR "PD L1 Inhibit*"[Title/Abstract] OR "Programmed Death-Ligand 1 Inhibit*"[Title/Abstract] OR "Programmed Death Ligand 1 Inhibit*"[Title/Abstract] OR "CTLA-4 Inhibit*"[Title/Abstract] OR "CTLA 4 Inhibit*"[Title/Abstract] OR "Cytotoxic T-Lymphocyte-Associated Protein 4 Inhibit*"[Title/Abstract] OR "Cytotoxic T Lymphocyte Associated Protein 4 Inhibit*"[Title/Abstract] OR "PD-1 Inhibit*"[Title/Abstract] OR "PD 1 Inhibit*"[Title/Abstract] OR "Programmed Cell Death Protein 1 Inhibit*"[Title/Abstract] OR "PD-1-PD-L1 Block*"[Title/Abstract] OR "PD 1 PD L1 Block*"[Title/Abstract] OR "Anti-PD-1"[Title/Abstract] OR "Anti PD 1"[Title/Abstract] OR "Anti-PD-L1"[Title/Abstract] OR "Anti PD L1"[Title/Abstract] OR "Anti-CTLA-4"[Title/Abstract] OR "Anti CTLA 4"[Title/Abstract] OR "Anti‐cytotoxic T‐lymphocyte‐associated protein 4"[Title/Abstract] OR "Pembrolizumab"[Title/Abstract] OR "Keytruda" [Title/Abstract] OR "Opdivo"[Title/Abstract] OR "Nivolumab"[Title/Abstract] OR "Ipilimumab"[Title/Abstract] AND (english[Filter]))) AND ("Gastrointestinal Microbiome*"[MeSH] OR "Gastrointestinal Microbiome*"[Text Word] OR "Gut Microbiome*"[Text Word] OR "Gut Microflora"[Text Word] OR "Gut Microbiota*"[Text Word] OR "Gastrointestinal Flora"[Text Word] OR "Gut Flora"[Text Word] OR "Gastrointestinal Microb*"[Text Word] OR "Gastrointestinal Microbial Communit*"[Text Word] OR "Gastrointestinal Microflora"[Text Word] OR "Gastric Microbiome*"[Text Word] OR "Intestinal Microbiome*"[Text Word] OR "Intestinal Microbiota*"[Text Word] OR "Intestinal Microflora"[Text Word] OR "Intestinal Flora"[Text Word] OR "Enteric Bacteria*"[Text Word] OR "Gastrointestinal Microbiome*"[Title/Abstract] OR "Gut Microbiome*"[Title/Abstract] OR "Gut Microflora"[Title/Abstract] OR "Gut Microbiota*"[Title/Abstract] OR "Gastrointestinal Flora"[Title/Abstract] OR "Gut Flora"[Title/Abstract] OR "Gastrointestinal Microb*"[Title/Abstract] OR "Gastrointestinal Microbial Communit*"[Title/Abstract] OR "Gastrointestinal Microflora"[Title/Abstract] OR "Gastric Microbiome*"[Title/Abstract] OR "Intestinal Microbiome*"[Title/Abstract] OR "Intestinal Microbiota*"[Title/Abstract] OR "Intestinal Microflora"[Title/Abstract] OR "Intestinal Flora"[Title/Abstract] OR "Enteric Bacteria*"[Title/Abstract] AND (english[Filter])) | **183** | **Search no. 1**  “Colonic Neoplasm” and synonyms  **AND**  **Search no. 2**  “Gut  Microbiom” and synonyms  **AND**  **Search no. 3**  Immune therapy and synonyms |
